# Nursing care for a patient with multiple organ fistulas and severe malnutrition-sarcopenia syndrome following surgery for irradiated rectal cancer: a case report

**DOI:** 10.3389/fmed.2026.1750366

**Published:** 2026-05-19

**Authors:** Changdi Li, Jing Zhang, Ai Li, Xing Zeng, Yang Yang

**Affiliations:** 1 Department of Gastrointestinal Oncology Surgery, Nanjing Jiangning Hospital, Nanjing, Jiangsu, China; 2 Department of Gastrointestinal Oncology Surgery, Nanjing Medical University Kangda College Jiangning School of Clinical Medicine, Nanjing, Jiangsu, China; 3 Nursing Department, Nanjing Jiangning Hospital, Nanjing, Jiangsu, China; 4 Chinese Hospital Reform and Development Institute, Nanjing University, Nanjing, Jiangsu, China; 5 Department of General Surgery, Jinling Hospital, Affiliated Hospital of Medical School, Nanjing University, Nanjing, Jiangsu, China

**Keywords:** case report, multidisciplinary team collaboration, radiation-induced enterovesical fistula, refined nursing management, severe malnutrition-sarcopenia syndrome

## Abstract

This case report presented a unique, integrated nursing management strategy for a 47-year-old male with extreme complexity arising from radiation-induced enterovesical fistula and severe malnutrition-sarcopenia syndrome following irradiated rectal cancer surgery, addressing a critical literature gap in systematic protocols for high-mortality cases. It demonstrated the practical application and sequencing of advanced, refined interventions within a Damage Control Surgery (DCS) framework, offering a replicable model. Key admission findings included severe metabolic derangement (BMI 13.2 kg/m^2^, serum albumin 20 g/L, handgrip strength 12 kg), uncontrolled intra-abdominal and gluteal abscesses with multidrug-resistant organisms, complex skin integrity issues (chronic sacrococcygeal pressure injury, incontinence-associated dermatitis), and anatomical complications (presacral abscess, small bowel dilation of 9.87 × 9.86 cm, right ureteric obstruction). Primary diagnoses were radiation-induced multivisceral fistula, severe malnutrition-sarcopenia, and intra-abdominal infection. A phased, MDT-driven approach included: infection control via dual-catheter negative pressure irrigation; nutritional rehabilitation using a stepwise protocol from parenteral to enteral nutrition (short-peptide formula) guided by precise energy calculation and managed with a “ten-aspect” strategy; advanced wound care combining recombinant human epidermal growth factor (rh-EGF) gel with silver ion dressings; and comprehensive functional rehabilitation through a personalized graded exercise prescription, including respiratory training via balloon blowing exercises enhanced by motivational tools (e.g., hanging inflated balloons bedside for engagement). Outcomes after 118 days showed substantial improvement: nutritional and metabolic recovery (weight increased from 38 kg to 41 kg, albumin rose to 35 g/L, electrolyte imbalances corrected); infection resolution (drainage cultures negative, abscesses resolved, bowel dilation reduced to 3.4 × 3.5 cm); physical function restoration (handgrip strength improved from 12 kg to 25 kg, patient progressed from bedbound to ambulating 2 h daily); and complete wound healing. The paramount lesson was that successful management required a systematic, phased, MDT-based nursing model integrating advanced technical interventions with meticulous, protocol-driven supportive care (e.g., structured nutrition and rehabilitation), proving critical for transforming prognosis in similar complex scenarios.

## 1 Introduction

Radiotherapy is a key treatment for colorectal malignancies and has significantly improved patient survival (1). However, pelvic irradiation can cause physical damage to the intestinal tract, referred to as radiation-induced intestinal injury (RII), which is categorized as acute or chronic based on the time of onset. Among the most serious complications of RII are enterocutaneous and related fistulas, occurring in approximately 7.5% of cases (2). These result from chronic inflammation and disruption of the intestinal barrier due to radiation exposure. The leakage of intestinal contents—such as digestive fluids, gas, and stool—through the fistula can lead to life-threatening conditions, including electrolyte imbalances, malnutrition, and systemic infections.

Radiation-related fistulas may present as entero-enteric, extra-intestinal, or enterocutaneous air fistulas. Based on anatomical location, they can be classified as small bowel fistulas, rectal fistulas, complex fistulas, or multivisceral fistulas—the latter involving adjacent organs such as the bladder or uterus (3). The pathophysiology is multifaceted, involving persistent polymicrobial infections, metabolic disturbances, progressive malnutrition, and multisystem dysfunction (4). For instance, the mixture of urine and intestinal contents often leads to recurrent abdominal and urinary tract infections, significantly impairing prognosis. Metabolic disorders arise from substantial intestinal fluid loss, resulting in hypoproteinemia and electrolyte imbalances, with hypokalemia occurring in over 60% of patients (4, 5). Nutritionally, patients frequently exhibit severe malnutrition (BMI < 18.5 kg/m^2^) and marked muscle wasting, exceeding 40% of normal values, which is strongly correlated with sarcopenia (6).

Conventional management remains challenging. Conservative approaches—such as bowel rest, parenteral nutrition (PN), and bladder irrigation—fail in up to 70% of cases (7). Although surgical repair is considered definitive, fistula recurrence rates remain high (30–50%) in malnourished patients with active abdominal infection (8). Recently, the concept of DCS has introduced a paradigm shift (9). This staged strategy—infection control → nutritional rehabilitation → definitive repair—has substantially reduced postoperative complications in high-risk patients (9). Specifically, continuous double-lumen irrigation under negative pressure (−80 to −120 mmHg) achieves infection control in 85% of cases (8), while stepwise nutritional support (total parenteral → combined enteral → full enteral nutrition) can raise albumin levels by 2.5-fold (4, 10).

Nursing care for patients with complex multivisceral fistulas is particularly challenging, especially when multiple organ systems are involved (4, 6). Challenges include simultaneous management of multi-catheter systems, complex wound and fistula care, severe malnutrition, refractory electrolyte imbalances, and comorbidities such as intestinal obstruction and pressure injuries (1, 5, 10). To date, systematic nursing protocols for multivisceral fistulas are lacking, particularly regarding standardized catheter care and stepwise nutritional transition. Thus, developing refined nursing strategies for such cases is of significant clinical relevance. In this report, we present a patient who was successfully managed through multidisciplinary treatment and meticulous nursing care. The following summarizes our nursing experience.

## 2 Clinical data

### 2.1 General information

A 47-year-old male (height: 170 cm; weight: 38 kg) was admitted on May 11, 2025, with a one-year history of a vesicointestinal fistula and a one-week history of recurrent abdominal distension and pain. His medical history was significant for rectal cancer, treated with radical resection in 2010 followed by adjuvant pelvic radiotherapy (20 sessions) and systemic chemotherapy. The patient denied any family history of malignancies or hereditary diseases. There was no known personal history of genetic disorders. He reported no prior history of psychiatric or psychological illnesses.

Upon admission, comprehensive risk assessments were performed. The patient scored 40 on the Morse Fall Scale, 17 on the Braden Scale (indicating moderate risk for pressure injury), and 4 on the VTE risk assessment. Self-care ability was severely compromised (score: 35). A catheter dislodgement risk score of 8 necessitated reinforced securement protocols. Nutritional assessment revealed significant risk (NRS 2002 score: 5) and compromised enteral tolerance (score: 5). Cognitive evaluation (MMSE score: 0) showed no notable psychological abnormalities.

Physical examination and initial workup revealed multiple concerns. The patient had an indwelling, clamped gastric tube (external length: 75 cm) and a urinary catheter draining purulent, debris-laden fluid. Skin assessment identified a chronic 3 × 6 cm pressure injury and a separate 1 × 1 cm Stage II pressure ulcer in the sacrococcygeal region, along with extensive perianal incontinence-associated dermatitis.

Admission laboratory tests showed progressive hypokalemia, refractory hypoproteinemia, and abnormal coagulation parameters (D-dimer: 5.05 mg/L), suggesting a hypercoagulable state. Imaging studies were pivotal: a fistulogram confirmed an active fistula tract with contrast extravasation from the posterior bladder wall (5 mm diameter, surrounded by cellulitis). A computed tomography (CT) scan revealed a presacral abscess (3.5 × 2.8 cm with air-fluid levels) and worsening right-sided hydronephrosis (22 mm) due to ureteric obstruction. Microbiological analysis of peritoneal and bladder irrigation fluids confirmed a multidrug-resistant infection, notably Methicillin-resistant *Staphylococcus aureus* (MRSA, +++).

Final Diagnoses on Admission: Status post rectal cancer resection; Vesicointestinal fistula (multivisceral type); Intra-abdominal and pelvic infection; Severe malnutrition with sarcopenia; Intestinal obstruction; Anemia; Gluteal abscess.

### 2.2 Diagnostic challenges

The diagnosis of this patient’s condition presented multiple, interconnected challenges that directly impacted management decisions.

1. Complex history and atypical presentation. The patient’s history of pelvic radiotherapy created a clinical scenario in which chronic radiation-induced injury was superimposed with acute complications. Presenting symptoms—recurrent abdominal pain, distension, and fever—were nonspecific, making it difficult to distinguish between an acute exacerbation of radiation enteritis, an intra-abdominal infection secondary to the fistula, or a developing mechanical obstruction. Imaging revealed significant small bowel dilation (9.87 × 9.86 cm), which required urgent differentiation between inflammatory ileus and true mechanical obstruction, as this distinction dictated whether to pursue initial conservative management or surgical intervention. Furthermore, while CT confirmed a fistula, severe surrounding tissue edema and active infection obscured the precise number of fistula tracts and their anatomical relationships.
2. Interference from multidrug-resistant infection. The confirmation of multidrug-resistant organisms (MDROs), including methicillin-resistant *Staphylococcus aureus* (MRSA) in peritoneal fluid, complicated the diagnostic process. The associated systemic inflammatory response could mask the underlying disease trajectory. More importantly, it elevated the risk of iatrogenic bacteremia from invasive diagnostic procedures (e.g., fistulography, cystoscopy), necessitating extreme caution. Consequently, the team relied more heavily on serial non-invasive imaging (e.g., dynamic CT) to assess progression and treatment response, which prolonged the diagnostic timeline and consumed significant resources.
3. Limited procedural tolerance due to critical debilitation. The patient’s extreme frailty upon admission—with severe malnutrition-sarcopenia syndrome (BMI 13.2 kg/m^2^, serum albumin 20 g/L, handgrip strength 12 kg)—severely limited tolerance for any diagnostic procedure. Lengthy imaging studies or invasive endoscopy, even with sedation, posed a high risk of cardiopulmonary compromise. This forced a diagnostic strategy that prioritized rapid, minimally invasive, and bedside-available techniques. While safeguarding the patient, this approach inevitably traded some degree of diagnostic comprehensiveness for safety, requiring the clinical team to continuously balance the need for information against the imperative of preventing iatrogenic harm.

### 2.3 Outcome

The patient received a comprehensive 118-day inpatient treatment following admission. Upon presentation with multiple organ fistulas, gluteal abscess, and intra-abdominal infection, the patient experienced recurrent high-grade fever during hospitalization. On May 11, dual-catheter negative pressure drainage combined with pulsed irrigation was initiated to manage the pelvic abscess (103 × 81 mm exudative lesion), intra-abdominal infection involving multidrug-resistant organisms, and gluteal abscess. Anti-infective therapy included intravenous linezolid 0.6 g every 12 h and meropenem 1 g every 8 h. A peripherally inserted central catheter (PICC) was placed to administer PN, including amino acid lipid emulsion (1,000 kcal), 5% glucose-sodium solution (500 mL), and albumin supplementation. Intravenous potassium was administered to correct hypokalemia and hypoalbuminemia. On May 13, enteral nutrition (EN) with a short-peptide formula (Peptamen) was initiated via nasogastric tube. On May 18, a triple-lumen catheter was inserted for continuous bladder irrigation to reduce fistula contamination. On June 8, an intestinal obstruction tube was placed for decompression due to incomplete intestinal obstruction. On July 29, a small bowel decompression tube was inserted percutaneously for further intestinal decompression.

Following the interventions, the patient’s nutritional status improved significantly: body weight increased from 38 kg to 41 kg, serum albumin rose from 20 g/L to 35 g/L, and handgrip strength improved from 12 kg to 25 kg. Infection markers also resolved, with drainage fluid cultures turning negative and fever subsiding. Fistula output decreased by 60%, and physical function recovered substantially—the patient progressed from being bedbound to ambulating 2 h daily at discharge, with gait speed increasing from 0 to 1.4 m/s over 6 meters. Pressure injuries and incontinence-associated dermatitis healed completely. Imaging showed resolution of small bowel dilation, with diameter reducing from 9.87 × 9.86 cm to 3.4 × 3.5 cm. The patient was discharged on September 7 with a nasogastric tube and urinary catheter in place for continued home-based nutrition and rehabilitation. Table 1 presents a timeline of significant clinical events, management actions, and patient outcomes.

**Table 1:** Timeline of clinical course and interventions.

Date	Clinical event or intervention	Intervention response	Patient outcome
May 11	Hospital admission with vesicointestinal fistula, abdominal infection, and severe malnutrition	Initiation of fasting, intravenous meropenem/linezolid, PN, and dual-catheter negative pressure drainage	Patient stabilized; initial control of systemic infection and metabolic derangement
May 13	–	Commencement of EN (Peptamen short-peptide formula) via nasogastric tube	Tolerance to EN established; foundational nutritional support initiated
May 18	–	Placement of a triple-lumen urinary catheter for continuous bladder irrigation	Fistula contamination reduced; urinary drainage optimized
May 27	Episode of fever	Administration of antipyretics and empirical antibiotic adjustment	Body temperature normalized within 24 h; infection markers showed progressive decline
June 8	Development of partial intestinal obstruction	Insertion of an intestinal obstruction tube for gastrointestinal decompression	Abdominal distension alleviated; intestinal motility partially restored
July 29	Worsening abdominal distension despite prior decompression	Percutaneous placement of a small bowel decompression tube under imaging guidance	Significant relief of abdominal distension; drainage of >2,000 mL intestinal fluid
August 9	Severe hypokalemia (2.4 mmol/L) detected	Intravenous potassium chloride supplementation initiated and titrated	Serum potassium levels normalized to 3.1 mmol/L within 12 h; electrolyte stability maintained
August 11	Significant abdominal distension recurred	Active decompression via intestinal tubes; drainage of 3,000 ml of fluid	Immediate relief of abdominal tension; patient reported improved comfort
August 16	High fever (38.9°C) with purulent exudate from right gluteal sinus tract	Initiation of targeted antibiotics (linezolid + meropenem); subcutaneous fine double-lumen tube irrigation and drainage	Fever resolved within 48 h; exudate volume decreased by 50%; gluteal infection controlled
August 31	Recurrent high fever (40.1°C)	Symptomatic management with antipyretics and re-evaluation of antibiotic therapy	Intermittent fever persisted but with reduced peak temperature; overall infection trend improved
September 2	Right gluteal sinus tract exudation significantly improved	Removal of subcutaneous fine drainage tube	Wound healing progressed; no signs of recurrent infection
September 7	Patient discharge planning	Discharge with nasogastric tube, pelvic double-lumen irrigation tube, and urinary catheter for home-based care	Weight increased from 38 kg to 41 kg; serum albumin rose to 35 g/L; capable of ambulating 2 h daily

## 3 Nursing plan

### 3.1 Establishment of multidisciplinary collaboration

A dedicated multidisciplinary team (MDT) was established to manage this complex case. The core team comprised general surgeons, urologists, radiologists, clinical microbiologists, a clinical pharmacist, a registered dietitian, wound/ostomy care specialists, and specialist nurses in nutrition and gastrointestinal surgery.

This team collaborated closely to guide all aspects of care. Key functions included: collaborative review of dynamic CT imaging by surgeons and radiologists to guide drainage tube placement; dynamic adjustment of anti-infective therapy by the surgical, microbiology, and pharmacy teams based on culture results; formulation and implementation of a stepwise nutrition plan by the dietitian and nutrition nurse; and specialized wound care led by the wound/ostomy therapist. Urologists contributed to diagnostic confirmation and treatment planning through cystoscopy and imaging. Regular imaging was used to assess fistula healing and adjust the irrigation regimen.

### 3.2 Control of multiple organ fistulas, gluteal abscess, and intra-abdominal infection

To achieve source control and mitigate systemic infection, a multi-modal management strategy was implemented, guided by the principle of damage control.

#### 3.2.1 Antimicrobial therapy and local care

Targeted anti-infective therapy with meropenem and levofloxacin was initiated based on antimicrobial susceptibility testing. Concurrently, meticulous local care of the perifistular skin was performed to prevent maceration and infection. This included prompt removal of intestinal fluid, timely dressing changes, the application of stoma powder, and the use of an infrared lamp to promote local skin dryness, a measure supported by clinical practice guidelines for managing exudating wounds (11).

#### 3.2.2 Early source control via drainage and pharmacologic support

The cornerstone of management was the establishment of early and effective drainage. A “peritoneal double-lumen tube” was placed for trans-anal irrigation and negative pressure suction of the enteric fistula effluent. A separate subcutaneous double-lumen tube was inserted to drain the perineal abscess. This approach of active irrigation combined with negative pressure drainage was prioritized to effectively remove purulent material and necrotic tissue, facilitating fistula tract cleansing. To reduce the volume of enteric output and promote a favorable environment for healing, intravenous somatostatin was continuously administered via an infusion pump for a planned course of 10–20 days, adhering to the established conservative management paradigm of “drainage, waiting, and reoperation” for enterocutaneous fistulas.

#### 3.2.3 Standardized nursing protocol for irrigation and drainage

A standardized nursing protocol was followed to ensure the safety and efficacy of the double-lumen tube systems. Key measures included: clear labeling of all tubing circuits; secure fixation of catheters to prevent dislodgement; and vigilant maintenance of system patency through regular flushing and checks. Negative pressure was carefully maintained within a therapeutic range of 10–20 kPa. The irrigation rate was titrated against the negative pressure to ensure effluent was promptly removed, thereby avoiding the dual risks of fluid accumulation (which could worsen infection) and excessive “dry suction” (which could traumatize the fragile sinus tract and cause bleeding) (12). To optimize drainage and patient comfort, positioning was managed with consideration of all indwelling tubes. As the patient had drainage tubes in the perineal/gluteal region and a small bowel decompression tube in the left lower abdomen, a left lateral position was primarily adopted to facilitate dependent drainage and prevent tube kinking or pressure, with repositioning scheduled every 2–4 h. The patient’s vital signs and systemic condition were closely monitored throughout.

### 3.3 Nutritional management

#### 3.3.1 Assessment, target calculation, and dynamic monitoring

Nutritional status was assessed according to the “three-step” approach. Initially, the NRS 2002 was used for risk screening, and the patient scored 5 points on the NRS 2002. Subsequently, malnutrition was evaluated using the Patient-Generated Subjective Global Assessment (PG-SGA) specifically designed for cancer patients, with a score of 11 points (indicating severe malnutrition). Finally, relevant nutritional indices were completed, including laboratory tests (albumin 20.8 g/L, prealbumin 39 g/L, hemoglobin 59 g/L, etc.), body composition measurements (appendicular skeletal muscle index 5.5 kg/m^2^), and physical function measurements (grip strength of the dominant hand 12 kg, unable to walk).

Initial calculation and principles: The daily energy target was first calculated by the clinical dietitian. The basal metabolic rate (BMR) was determined using the Harris-Benedict equation: 66.47 + (13.75 × weight in kg) + (5.0033 × height in cm) - (6.775 × age). The BMR was then multiplied by standard activity (1.2), stress (1.3), and temperature (1.2) factors for critically ill patients (13). Protein was targeted at 1.5–2.0 g/kg/day. Dynamic adjustment: This calculated target was not static; it served as a baseline and was dynamically reassessed and adjusted throughout the treatment course based on the patient’s evolving clinical status, metabolic response, and tolerance to nutrition support. Given a serum 25-hydroxyvitamin D level <50 nmol/L, vitamin D supplementation (600–800 IU/d) was also initiated (14–17).

#### 3.3.2 Implementation of a phased, protocol-driven strategy

A damage control-based, phased nutritional strategy was rigorously implemented. The initial phase focused on parenteral stabilization: total parenteral nutrition (TPN) was delivered via a peripherally inserted central catheter (PICC) to urgently address severe metabolic derangements and nutritional deficits. This was followed by the early initiation and progressive advancement of EN. Despite the formidable challenges of concurrent intestinal obstruction, an active enteric fistula, and ongoing gastrointestinal decompression, the clinical team opted for early trophic EN. Beginning on May 13, a short-peptide formula (Peptamen) was administered via a nasoenteral tube at a rate of 20 mL/h to maintain gut mucosal integrity. The subsequent, meticulous advancement of EN was guided by the structured “Ten-Aspect” management protocol (Table 2), which ensured safe, incremental increases in delivery tailored to the patient’s tolerance and complex clinical status.

**Table 2:** “Ten-Aspect” management protocol.

Aspect	Key operational examples
Tolerance	Assessed every 4–6 h via a dedicated scale. Rate was adjusted or EN suspended based on score (0–2: continue/advance; 3–4: slow and reassess; ≥5: stop and intervene)
Angle	Head-of-bed elevated to 30–45°
Temperature	EN solution maintained at 37–40 °C
Rate	Constant infusion, starting at 10–20 mL/h, gradually increased, maximum ≤70 mL/h
Concentration	Gradually increased from low to high
Cleanliness	Aseptic preparation; opened solutions refrigerated
Comfort	Patient’s subjective feelings and adverse reactions actively managed
Patency	Tube flushed with 20–30 mL warm water/saline before/after feeds and every 2–4 h
pH	Peptamen (pH 4.45) selected for compatibility with proximal small intestine acidity
Saturation	Stepwise increase in concentration (started at 10%), adjusted dynamically

### 3.4 Gluteal pressure injury and incontinence-associated dermatitis care

#### 3.4.1 Gluteal pressure injury care

Upon admission, improperly managed gluteal pressure injuries with adhered gauze were addressed. After gentle removal with saline-moistened gauze, the wounds were irrigated with sterile saline. A specialized care plan was established in consultation with a wound care specialist. Following debridement, rh-EGF was applied to the wound bed. A silver-ion dressing was then placed over the ulcer, with at least a 1 cm perimeter margin, to manage bioburden. A transparent film dressing was secured around the silver dressing to protect it from moisture and contamination from the adjacent fistula. Dressings were changed every 2 days or immediately if soiled, with the frequency gradually extended as the wounds improved. The rationale for this combined topical regimen was based on evidence that rh-EGF promotes epithelialization and fibroblast proliferation (18–20), while silver ions provide broad-spectrum antimicrobial activity without significantly inhibiting growth factor function, thereby creating a conducive environment for healing (21–25). By discharge, all pressure injuries had completely healed.

#### 3.4.2 Gluteal incontinence-associated dermatitis care

**Figure 1:**
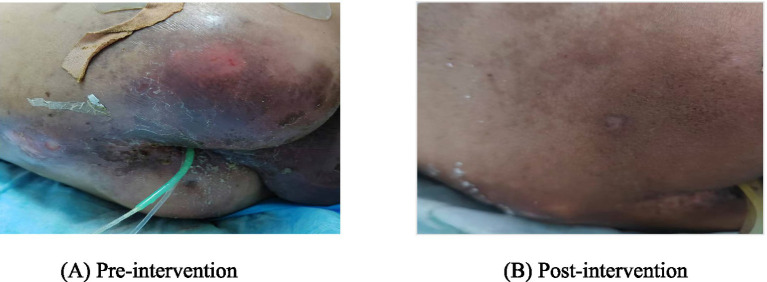
Comparison of sacral pressure injury and incontinence-associated dermatitis before and after treatment. **(A)** Pre-intervention. **(B)** Post-intervention.

The perianal skin affected by incontinence-associated dermatitis was managed with a protective barrier strategy. The area was cleansed with sterile normal saline and patted dry. A layered barrier was then constructed by first applying stoma powder to absorb moisture, followed by a spray-on antimicrobial film-forming dressing. This two-step process was repeated once daily. The stoma powder acted as a physical moisture-absorbing barrier, while the antimicrobial dressing provided protection against microbial colonization (26–30). This regimen, emphasizing gentle cleansing and robust skin protection, resulted in the complete resolution of the dermatitis by the time of discharge. At the time of discharge, the patient’s sacral pressure injury and incontinence-associated dermatitis had completely resolved (Figure 1).

### 3.5 Intestinal obstruction care

#### 3.5.1 Intestinal obstruction tube management

For the management of partial intestinal obstruction, an intestinal obstruction tube was placed. Pre-insertion safety was ensured by verifying the guidewire did not protrude beyond the catheter tip to prevent visceral injury (31). Post-insertion care involved confirming tube position, inflating the balloon with sterile water, and securing the tube with a nasal sticker, leaving sufficient length to allow for peristaltic advancement. The patient was maintained in a semi-Fowler’s position to facilitate drainage and prevent tube kinking. Insertion length and drainage were monitored closely. The tube was removed after radiographic confirmation of obstruction resolution.

#### 3.5.2 Small bowel puncture drainage tube care

**Figure 2:**
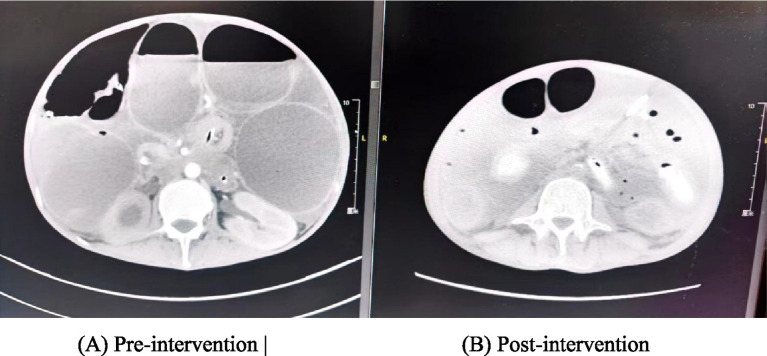
Comparative abdominal CT scans before and after treatment. **(A)** Pre-intervention. **(B)** Post-intervention.

For concurrent, more distal obstruction, a percutaneous small bowel drainage tube was placed as a minimally invasive alternative. Nursing care focused on optimizing drainage: the patient was primarily positioned in left lateral decubitus to facilitate gravity-dependent drainage and repositioned regularly. Drainage efficacy and tube function were vigilantly monitored; a sudden decrease in output or a change in odor prompted assessment for displacement or new fistula formation. The treatment’s success was objectively evaluated by serial imaging, which showed a resolution of the severe small bowel dilation (reduced from 9.87 × 9.86 cm to 3.4 × 3.5 cm) (Figure 2).

### 3.6 Graded exercise program

A 14-day personalized graded exercise prescription was implemented to address severe sarcopenia. Given the patient’s critical initial state, all exercises, especially during the early phase upon admission, were conducted under continuous electrocardiographic (ECG) monitoring for safety. Exercise intensity was titrated to induce mild fatigue, with predefined termination criteria: exercise was immediately terminated if the patient’s heart rate exceeded 70% of the baseline or dropped by 20%, systolic blood pressure exceeded 180 mmHg or dropped by 20%, or pulse oximetry saturation fell below 90% (Table 3).

**Table 3:** Exercise program.

Exercise item	Phase 1 (Days 1–3)	Phase 2 (Days 4–7)	Phase 3 (Days 8–14)
Balloon blowing exercises	10 balloons/d	20 balloons/d	30 balloons/d
Grip strengthening	10 reps × 3/d	30 reps × 3/d	50 reps × 4/d
Arm flexion	20 reps × 3/d	50 reps × 3/d	50 reps × 4/d
Knee flexion	20 reps × 3/d	30 reps × 3/d	50 reps × 4/d
Foot pump exercise	30 reps × 3/d	40 reps × 3/d	50 reps × 4/d
Pedaling exercise	30 reps × 3/d	40 reps × 3/d	50 reps × 4/d
Sitting in bed	15 min × 3/d	30 min × 3/d	60 min × 4/d
Deep breathing	10 reps × 3/	20 reps × 3/d	30 reps × 4/d
Gastrocnemius squeezing	5 min × 3/d	7 min × 3/d	10 min × 4/d
Bridge exercises	–	10 reps × 2/d	10 reps × 2/d
Pull-ups	–	–	10 reps × 2/d
Standing by bedside	–	–	10 min × 1–2/d

The volume of balloon blowing was maintained or gradually increased after Day 14 based on tolerance, with a maximum not exceeding 60 balloons per day. The patient progressively transitioned to walking during Phase 3.

**Figure 3:**
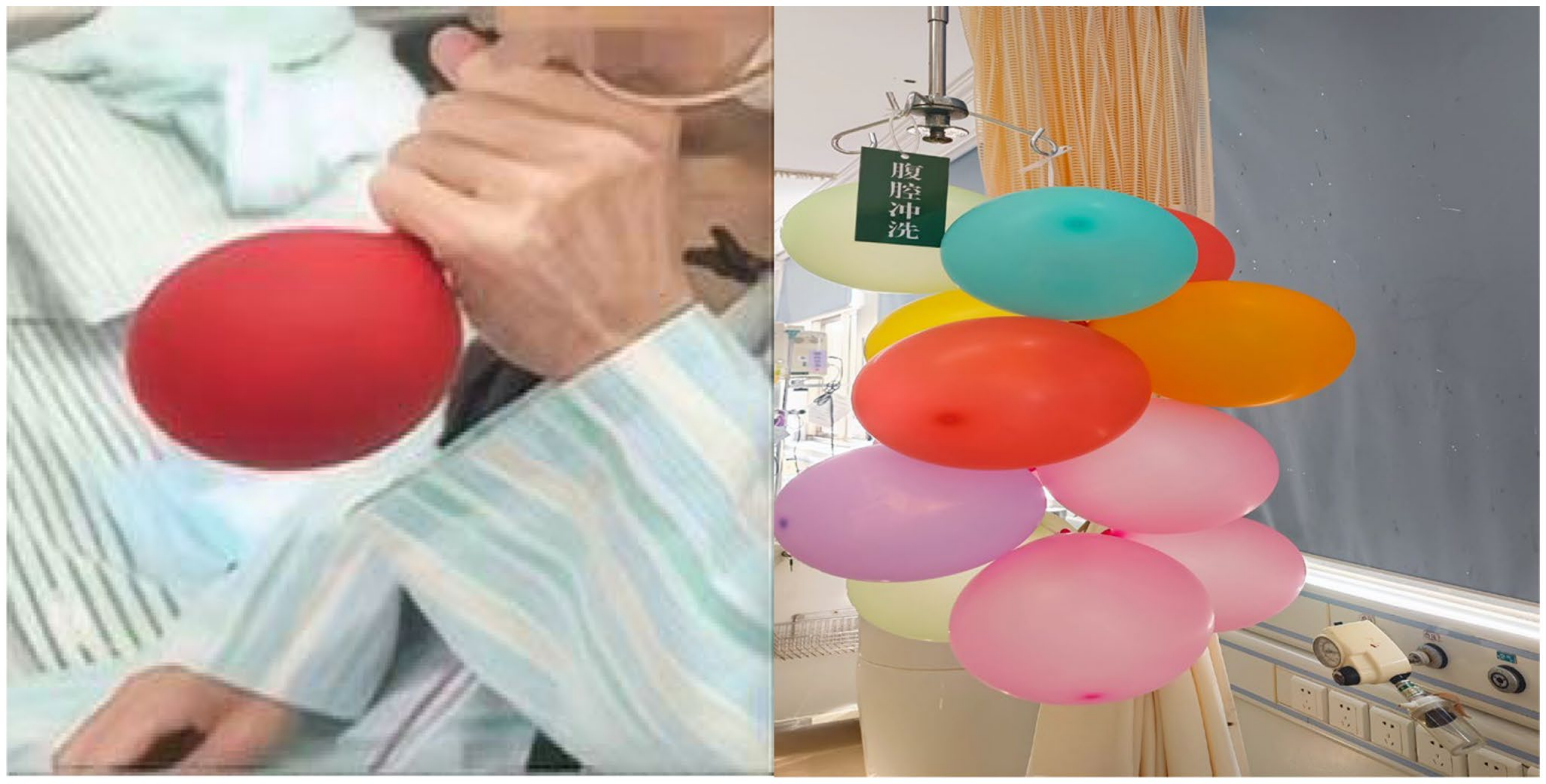
A symbol of achievement: Balloons as a simple tool to enhance rehabilitation morale.

The prescription followed a structured, three-phase progression (32):

To enhance motivation and adherence, a balloon inflated during the daily respiratory training was hung at the bedside as a visual progress marker (Figure 3). This monitored, graded rehabilitation was integral to recovery. By discharge, the patient’s grip strength improved from 12 kg to 25 kg, and he progressed from being bedbound to independently ambulating for 2 h daily.

## 4 Patient-reported outcomes

During the treatment course, the patient’s subjective experiences and feedback regarding key nursing interventions were gathered through informal interviews:

Motivation and engagement in rehabilitation: The patient reported that the graded exercise program, particularly the simple intervention of hanging the daily inflated balloon at the bedside as a visual progress marker, positively impacted his morale and engagement. He described this practice as “transforming rehabilitation from a task into a motivating ritual,” which alleviated feelings of demoralization and actively encouraged his adherence to the training.

Understanding of and collaboration in complex therapy: The patient provided feedback that the detailed explanations from the nursing team regarding the “Ten-Aspect” enteral nutrition management protocol enhanced his trust in the treatment plan. The dynamic adjustments made based on his tolerance feedback made him feel like a collaborator in the process, thereby reducing discomfort and strengthening clinician-patient collaboration.

Wound care and psychological support: The patient indicated that the professional wound care for pressure injuries and dermatitis, coupled with the meticulousness and empathy demonstrated by the nurses during procedures, significantly reduced his pain and fear of permanent tissue damage, accelerating his psychological adaptation.

Overall meaning of recovery: The patient identified “the transition from being completely bedbound to independent walking at discharge” as the most significant outcome. He viewed the consistent and clear communication from the multidisciplinary team as crucial in addressing his integrated physical and psychological care needs, which was foundational in supporting him through the prolonged and challenging treatment period.

## 5 Prognosis

he prognosis for this patient is assessed as follows, based on the clinical course and established literature: (1) Short-to-medium term prognosis is guardedly optimistic: The significant improvements achieved during the 118-day integrated inpatient care—including controlled infection, markedly improved nutritional status (weight increase from 38 kg to 41 kg, serum albumin from 20 g/L to 35 g/L), and restored physical function (progressing from bedbound to ambulating 2 h daily)—provide a solid foundation for a positive short-term outcome. However, discharge with a nasogastric tube and urinary catheter indicates that the patient remains in a stable yet fragile “damage control” phase. The key prognostic indicators for the next 3–6 months will include: evidence of fistula tract closure or reduction in output, absence of recurrent intestinal obstruction, sustained nutritional autonomy or successful home-based nutritional support, and continued functional gains. (2) Long-term prognosis retains significant risks requiring vigilant monitoring: The long-term outlook is complicated by the irreversible nature of radiation-induced tissue damage. The primary long-term risks include: ① Fistula recurrence or development of new fistulas: The radiated bowel and bladder walls remain friable and have poor healing capacity. Even with current stabilization, the risk of fistula recurrence under stress or the development of new fistulas at other sites remains substantial, with literature citing recurrence rates of 30–50% in malnourished patients with active infection. ② Chronic bowel dysfunction: The underlying radiation enteritis predisposes the patient to long-term complications such as persistent dysmotility, malabsorption, and chronic partial obstruction. ③ Lifelong management of malnutrition-sarcopenia syndrome: Complete reversal of severe sarcopenia is a prolonged process. The patient will require long-term, meticulous nutritional support and a structured exercise regimen to maintain quality of life, prevent further functional decline, and reduce the risk of subsequent infections. (3) Determinants of favorable prognosis: A positive long-term outcome is highly dependent on continuous, coordinated follow-up within a MDT framework. This includes regular imaging assessments, individualized adjustments to nutritional support, and a progressive rehabilitation plan. Crucially, the patient’s and family’s adherence to and competence in managing home-based care (e.g., tube care, enteral feeding) will be a pivotal factor influencing overall survival and quality of life. The refined nursing model demonstrated in this case, if sustained post-discharge, offers a valuable framework for optimizing this patient’s long-term prognosis.

## 6 Transferability and generalizability of the nursing care model

The integrated, DCS-guided nursing model presented in this case report consists of both broadly applicable frameworks and elements specific to this patient’s extreme condition. Distinguishing these is crucial for clinical adoption.

**Transferable core frameworks and principles**:

DCS phased approach: The overarching “control (infection) → rebuild (nutrition) → restore (function)” sequence is a universal strategy for managing any critically ill, hypermetabolic patient, such as those with major trauma, complex abdominal sepsis, or post-major surgery complications.

MDT coordination structure: The established framework for collaboration between surgery, nursing, nutrition, pharmacy, radiology, and wound care specialists is a transferable operational model. The principles of regular, structured communication and shared decision-making are key takeaways.

Structured protocol application: The “ten-aspect” EN management protocol’s primary value lies in its systematic monitoring and adjustment framework. Other institutions can adopt this checklist approach, adapting the specific parameters (e.g., tolerance scale used, flushing standards) to local resources.

Early, personalized rehabilitation: The principle of initiating graded, personalized exercise prescriptions early in the course of severe illness to combat sarcopenia and improve nutritional utilization is a key, generalizable takeaway.

Non-pharmacological motivational strategy: The use of simple, visual progress markers (e.g., the displayed balloon) is a low-cost, highly feasible intervention to improve patient engagement in rehabilitation, easily replicable across different settings.

**Case-specific elements requiring contextual adaptation**:

Specific technical parameters: The precise anti-infective regimen (e.g., meropenem + linezolid for MRSA) and the meticulously calculated energy target (2098.72 kcal/day) must be re-evaluated for each patient based on microbiology, indirect calorimetry (if available), and resources.

Concurrent management of extreme device burden: The simultaneous care of seven indwelling tubes (e.g., dual-catheters, bladder irrigation, bowel decompression) reflects this case’s unique complexity. While the number of devices may vary, the *standardized care principles* for each (secure fixation, patency, asepsis) remain applicable.

Specific product combinations: The use of *rh-EGF gel + silver-ion dressings* was a product of local availability and wound assessment. The transferable concept is the “*combination of a bio-active healing agent with an anti-microbial dressing*” for complex wounds, not the specific brand.

## 7 Limitations

As a detailed single-case report, this study has inherent limitations that must be considered when interpreting the findings. Study design: The lack of a control group prevents the establishment of causality. We can describe a temporal association between the interventions and the positive outcomes, but we cannot conclusively prove that the nursing model was the sole or primary cause of recovery. Generalizability: The outcomes described here are not generalizable to all patients with radiation-induced multivisceral fistulas. The extreme presentation, specific comorbidities, and individual patient response are unique. This report serves as a “proof-of-concept” and a detailed roadmap, not a guarantee of identical results. Dependence on specialized resources: Successful implementation of this model relied heavily on the resources of a tertiary care center, including continuous, on-demand availability of a mature MDT, specialist nurses, and advanced interventional radiology. Full replication may be challenging in resource-constrained settings. Potential for reporting bias: As the clinical team involved in the patient’s care, the authors may have an inherent bias toward reporting the interventions and outcomes in a positive light.

## Data Availability

The original contributions presented in the study are included in the article/supplementary material, further inquiries can be directed to the corresponding authors.
